# Salivary Dysfunction in Primary and Secondary Sjögren’s Syndrome: Functional Assessment and the Exploratory Role of Salivary Microcrystallization

**DOI:** 10.3390/dj14080476

**Published:** 2026-08-03

**Authors:** Cristina-Angela Ghiorghe, Ionuț Tărăboanţă, Cristina Iordache, Codrina Ancuţa, Alexandru Lodbă, Claudiu Topoliceanu, Mihaela Sălceanu, Gianina Iovan, Irina Nica, Galina Pancu

**Affiliations:** Grigore T. Popa University of Medicine and Pharmacy, 16 Universitatii Str., 700115 Iasi, Romania; cristina.ghiorghe@umfiasi.ro (C.-A.G.); marina.iordache@umfiasi.ro (C.I.); codrina.ancuta@umfiasi.ro (C.A.); lodba.alexandru@d.umfiasi.ro (A.L.); claudiu.topoliceanu@umfiasi.ro (C.T.); mihaela.salceanu@umfiasi.ro (M.S.); gianina.iovan@umfiasi.ro (G.I.); nica.irina@umfiasi.ro (I.N.); galina.pancu@umfiasi.ro (G.P.)

**Keywords:** Sjögren’s syndrome, salivary dysfunction, unstimulated salivary flow, stimulated salivary flow, salivary pH, salivary microcrystallization, IMK

## Abstract

**Background**: Salivary gland dysfunction is a central manifestation of Sjögren’s syndrome, but the relationship between quantitative salivary impairment and salivary microcrystallization remains insufficiently defined. This study aimed to assess unstimulated and stimulated whole salivary flow and salivary pH in patients with primary and secondary Sjögren’s syndrome and to explore salivary microcrystallization indices as complementary descriptive markers of salivary physicochemical patterns. **Materials and Methods**: This cross-sectional observational study included 126 patients diagnosed with primary or secondary Sjögren’s syndrome. Unstimulated whole salivary flow, stimulated whole salivary flow, and salivary pH were assessed under standardized conditions. Salivary dysfunction severity was classified according to predefined functional salivary parameters. Salivary microcrystallization was evaluated microscopically on dried saliva samples and expressed using the IMK-RFR and IMK-RFS indices. Differences between disease subtypes and associations between salivary parameters, microcrystallization indices, and dysfunction severity were analyzed. **Results**: The mean unstimulated salivary flow rate was 0.272 ± 0.246 mL/min, while the mean stimulated salivary flow rate was 1.04 ± 0.50 mL/min. In unadjusted analyses, patients with primary Sjögren’s syndrome showed lower unstimulated and stimulated salivary flow rates than patients with secondary Sjögren’s syndrome. Both salivary flow parameters were inversely associated with salivary dysfunction severity. In an exploratory internal consistency ROC analysis, unstimulated salivary flow showed good internal agreement with grade ≥ 3 salivary dysfunction, while stimulated salivary flow showed very high internal agreement. Salivary pH showed limited internal consistency with dysfunction severity. Firth penalized logistic regression indicated that stimulated whole salivary flow remained associated with grade ≥ 3 salivary dysfunction after adjustment for unstimulated salivary flow. IMK values described salivary microcrystallization patterns within the Sjögren’s syndrome cohort; however, they were not robustly correlated with salivary flow, pH, or dysfunction severity. **Conclusions**: Salivary flow assessment remains a simple, non-invasive, and clinically relevant method for evaluating glandular dysfunction in Sjögren’s syndrome. In this cohort, primary Sjögren’s syndrome was associated with lower salivary flow rates, but these unadjusted findings should be interpreted as descriptive. Salivary microcrystallization provided exploratory information on qualitative salivary patterns, but its clinical value remains limited and requires methodological standardization and validation in larger prospective studies.

## 1. Introduction

Sjögren’s syndrome is a systemic autoimmune disease characterized by predominant involvement of the exocrine glands, particularly the salivary and lacrimal glands, although systemic manifestations may affect multiple organs and tissues [1]. The clinical picture is dominated by sicca symptoms, especially xerostomia and keratoconjunctivitis sicca, but fatigue, musculoskeletal pain, and extraglandular manifestations are also frequently reported. Clinically, Sjögren’s syndrome may occur as a primary disease or in association with other systemic autoimmune disorders, traditionally referred to as secondary Sjögren’s syndrome [2,3,4].

Salivary gland dysfunction is one of the central manifestations of the disease and has direct consequences for oral health and quality of life. Reduced salivary secretion impairs mucosal lubrication, buffering capacity, antimicrobial protection, and dental hard tissue defense, thereby increasing the risk of dysphagia, dental caries, oral infections, mucosal discomfort, and impaired daily functioning [5]. Objective assessment of salivary hypofunction is therefore clinically relevant. Among the available methods, unstimulated whole salivary flow and stimulated whole salivary flow remain simple, non-invasive, and widely used functional tests [6]. In the 2016 ACR/EULAR classification criteria for primary Sjögren’s syndrome, an unstimulated whole salivary flow rate of ≤0.1 mL/min is included as an objective criterion, while stimulated salivary flow may provide complementary information regarding the residual secretory capacity of the salivary glands [7,8]. Salivary gland scintigraphy provides dynamic, gland-specific assessment of radiotracer uptake and excretion in the major salivary glands. However, because of radiation exposure, limited availability, and the growing use of salivary gland ultrasonography, it is considered a complementary rather than routine investigation [9].

Despite the clinical utility of salivary flow measurement, several aspects remain insufficiently clarified. Differences between primary and secondary Sjögren’s syndrome are commonly discussed in relation to systemic autoimmune background but less frequently through direct comparison of measurable salivary gland dysfunction. Moreover, conventional salivary parameters mainly describe the quantitative component of secretion, whereas qualitative salivary changes are less routinely captured [10]. This is relevant because saliva is a complex biological fluid containing electrolytes, proteins, glycoproteins, enzymes, and immune mediators, and its physical and biochemical properties may be altered in autoimmune glandular disease [11]. Therefore, additional non-invasive parameters able to reflect qualitative salivary changes could provide complementary information beyond salivary flow alone [12,13].

Salivary microcrystallization represents a potential exploratory approach for evaluating the physicochemical organization of dried saliva. The morphology of crystallization patterns may indirectly reflect variations in salivary composition, including electrolyte balance and macromolecular content [14]. Previous research on saliva and other biological fluids has suggested that desiccation patterns may be influenced by systemic and local biological conditions. However, the clinical significance, reproducibility, and standardization of salivary microcrystallization in Sjögren’s syndrome remain insufficiently validated. Importantly, salivary microcrystallization is not included in current classification criteria or routine management recommendations for Sjögren’s syndrome; therefore, its potential value should be interpreted as exploratory rather than diagnostic [15,16].

In this context, the present study aimed to evaluate salivary dysfunction in patients with primary and secondary Sjögren’s syndrome by assessing unstimulated whole salivary flow, stimulated whole salivary flow, and salivary pH. A secondary exploratory objective was to describe salivary microcrystallization indices and to examine their possible relationships with functional salivary parameters, disease subtype, and salivary dysfunction severity. IMK was therefore considered a complementary exploratory parameter, not a diagnostic test or an independent marker of salivary dysfunction severity.

## 2. Materials and Methods

### 2.1. Study Design and Participants

This cross-sectional observational study was conducted between August 2024 and March 2026 at the Clinical Rehabilitation Hospital in Iași, Romania. The study included 126 consecutive patients diagnosed with Sjögren’s syndrome and evaluated in a clinical setting.

Patients were classified as having primary or secondary Sjögren’s syndrome based on clinical, serological, and objective glandular assessment. Primary Sjögren’s syndrome was defined according to the 2016 ACR/EULAR [9] classification criteria, using available items such as anti-SSA/Ro antibody status, minor salivary gland biopsy when available, ocular tests, and unstimulated whole salivary flow. Secondary Sjögren’s syndrome was defined as objective sicca manifestations and salivary gland involvement occurring in patients with a previously diagnosed systemic autoimmune disease, such as rheumatoid arthritis, systemic lupus erythematosus, systemic sclerosis, mixed connective tissue disease, or other autoimmune rheumatic diseases. Alternative causes of salivary hypofunction were excluded when considered clinically relevant.

Patients were eligible for inclusion if they were adults diagnosed with primary or secondary Sjögren’s syndrome and had complete clinical and salivary assessment data. Exclusion criteria included conditions or factors that could independently influence salivary secretion, such as previous head and neck radiotherapy, active oral infections, uncontrolled diabetes mellitus, dehydration, pregnancy, current smoking, and use of medications with relevant xerogenic effects, including anticholinergics, antidepressants, antihistamines, diuretics, or other drugs considered clinically relevant at the time of evaluation.

For each participant, demographic, clinical, biological, and salivary parameters were recorded at the time of evaluation. The main variables analyzed in the present study were age, sex, type of Sjögren’s syndrome, unstimulated whole salivary flow, stimulated whole salivary flow, salivary pH, severity grade of salivary dysfunction, and salivary microcrystallization indices.

The study protocol was approved by the Ethics Committee of the Clinical Rehabilitation Hospital in Iași, Romania (approval no. 426/7 April 2024), and the study was conducted in accordance with the principles of the Declaration of Helsinki. Written informed consent was obtained from all participants before inclusion.

**Figure 1:**
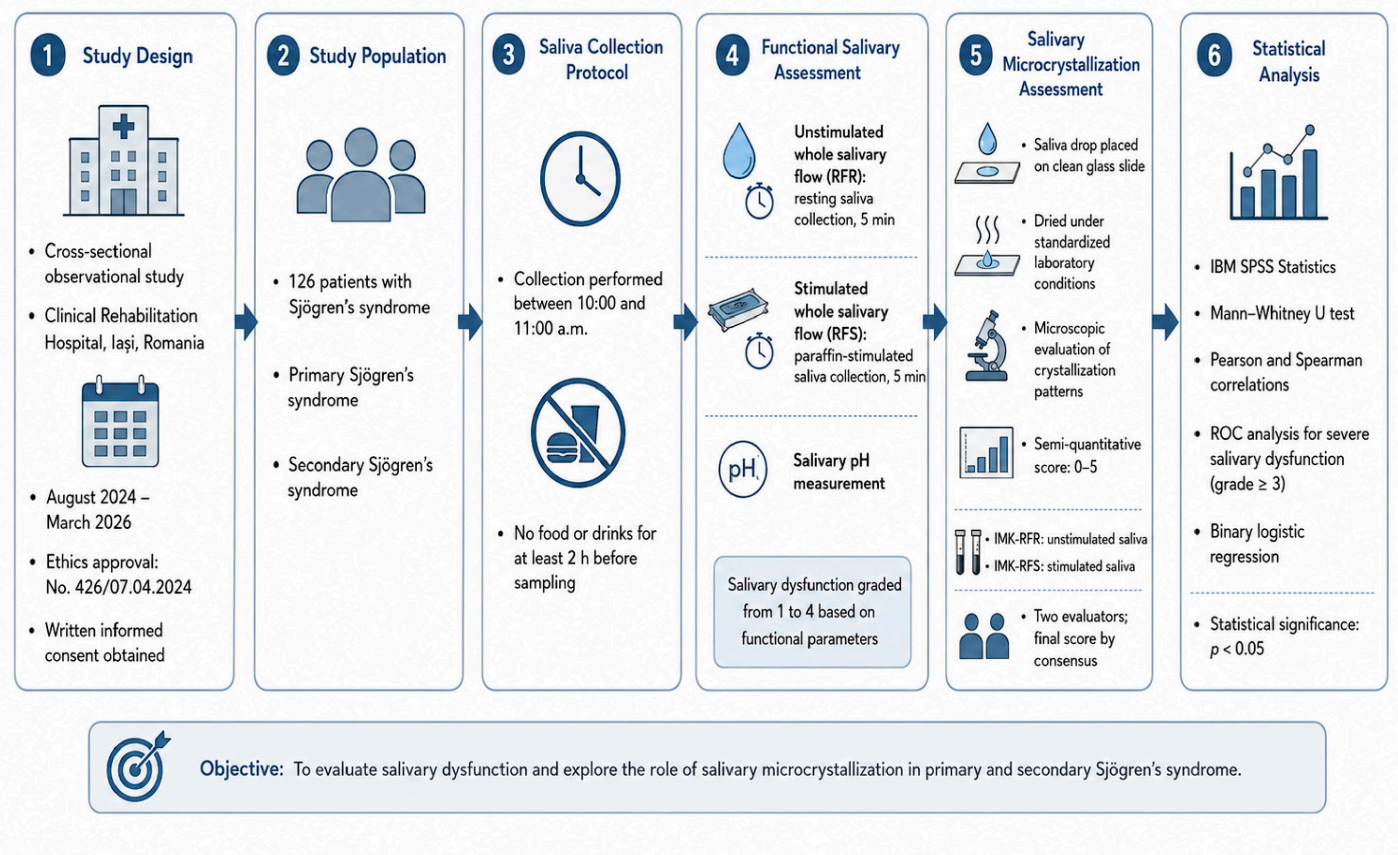
**Overview of all stages of the study protocol.**

The complete sequence of the study, from participant inclusion and saliva collection to functional, microcrystallization, and statistical analyses, is illustrated in Figure 1.

### 2.2. Salivary Functional Assessment

Salivary gland function was assessed by measuring unstimulated whole salivary flow and stimulated whole salivary flow. Salivary testing, including salivary pH assessment, was performed using the Saliva-Check BUFFER kit (GC Europe N.V., Leuven, Belgium; lot no. 2305116), according to the manufacturer’s instructions. To reduce circadian variability, all saliva samples were collected between 10:00 and 11:00 a.m. Participants were instructed not to eat, drink, smoke, or perform oral hygiene procedures for at least two hours before saliva collection.

Unstimulated whole saliva was collected under resting conditions over a period of 5 min and expressed as mL/min. Stimulated whole saliva was subsequently collected over a period of 5 min after paraffin chewing stimulation and was also expressed as mL/min. All salivary flow assessments were performed using the same protocol throughout the study.

Salivary dysfunction severity was classified using a predefined four-grade scale based on functional salivary parameters, including unstimulated whole salivary flow, stimulated whole salivary flow, and salivary pH. Grade 1 indicated mild salivary dysfunction, grade 2 moderate dysfunction, grade 3 moderate-to-severe dysfunction, and grade 4 severe dysfunction. The thresholds used for each grade are presented in Table 1.

### 2.3. Salivary Microcrystallization Assessment

Salivary microcrystallization was evaluated as an exploratory parameter using the salivary microcrystallization index, according to the method of P.A. Leus, modified after L.V. Belskaya [16]. The method is based on the microscopic assessment of the crystallization pattern obtained after complete dehydration of a saliva drop placed on a clean glass slide. Previous applications of this method classify IMK values as high, medium, or low microcrystallization, using the following intervals: 0.6–1.0, 0.4–0.6, and 0–0.4, respectively.

In the present study, unstimulated and stimulated whole saliva samples collected during the functional salivary assessment were used for IMK evaluation. For each sample, a standardized saliva drop was placed on a clean, dry glass slide and allowed to dry completely at room temperature, under stable laboratory conditions, without forced ventilation, heating, or direct sunlight. After complete dehydration, the dried saliva drop was examined using a digital optical microscope, Optika B-190TB, 1000×, Optika S.r.l., Ponteranica, Italy, at 20× magnification.

The microcrystallization pattern was assessed in the central area of the dried saliva drop, where crystalline structures are most representative. The IMK was calculated as the ratio between the number of counting-grid points projected onto crystalline structures and the total number of grid points projected onto the entire analyzed field of the dried saliva drop. Higher IMK values indicated more preserved arborescent or fern-like crystalline organization, whereas lower IMK values indicated poorly represented, diffuse, fragmented, conglomerated, or needle-like crystallization patterns.

Two indices were recorded: IMK-RFR, corresponding to unstimulated saliva samples, and IMK-RFS, corresponding to stimulated saliva samples. All slides were evaluated independently by two blinded examiners. In cases of disagreement, the final value was established by consensus. Inter-observer reproducibility was assessed using the intraclass correlation coefficient. IMK values were not used to define Sjögren’s syndrome subtype or salivary dysfunction severity and were interpreted only as exploratory descriptors of dried saliva microcrystallization patterns.

The interpretation of salivary microcrystallization index values was based on the Leus method, modified after Belskaya/Belischaia, using a three-level classification of IMK values, as presented in Table 2.

### 2.4. Statistical Analysis

Statistical analyses were performed using IBM SPSS Statistics, version 29.0.0., IBM Corp., Armonk, NY, USA. Continuous variables were expressed as mean ± standard deviation or as median and interquartile range, depending on data distribution. Categorical variables were reported as absolute frequencies and percentages.

Sample size adequacy was assessed using G*Power software, version 3.1.9.7. The calculation was based on the main comparison between patients with primary and secondary Sjögren’s syndrome, using unstimulated whole salivary flow as the primary functional outcome. Assuming a two-sided α level of 0.05, 80% statistical power, and the observed allocation ratio between groups, the minimum required sample size was approximately 121 patients. Therefore, the final cohort of 126 patients was considered adequate for the main salivary flow comparison. Analyses involving salivary microcrystallization indices were considered exploratory.

Comparisons between primary and secondary Sjögren’s syndrome were performed using the Mann–Whitney U test and were considered exploratory and unadjusted. Because the secondary Sjögren’s syndrome group was clinically heterogeneous, these results were interpreted as descriptive cohort-level differences rather than independent disease-subtype effects. Associations between salivary parameters, microcrystallization indices, and clinical variables were assessed using Pearson and Spearman correlation coefficients, as appropriate.

For between-group comparisons, effect sizes were reported together with 95% confidence intervals whenever appropriate. Median differences were estimated using the Hodges–Lehmann estimator with 95% confidence intervals. For ROC analyses, AUC values were reported with 95% confidence intervals, calculated using the DeLong method. Correlation coefficients were accompanied by 95% confidence intervals when applicable. Logistic regression results were reported as odds ratios with 95% confidence intervals.

Receiver operating characteristic curve analysis was performed as an exploratory internal consistency assessment to evaluate how unstimulated whole salivary flow, stimulated whole salivary flow, and salivary pH aligned with the predefined salivary dysfunction grading system. Because salivary dysfunction severity was derived from these functional parameters, ROC results were not interpreted as independent diagnostic or predictive validation.

Inter-observer agreement for IMK evaluation was assessed in a randomly selected subset of 30 samples independently scored by two blinded evaluators. Agreement was evaluated using the intraclass correlation coefficient for absolute agreement.

Because multiple exploratory correlations were performed for the salivary microcrystallization indices, *p*-values from the Spearman correlation analyses were additionally adjusted using the Holm–Bonferroni method. Both nominal and adjusted *p*-values were considered in the interpretation of IMK-related associations.

Binary logistic regression analysis was performed to identify factors associated with grade ≥ 3 salivary dysfunction. Unstimulated and stimulated whole salivary flow rates were included as independent variables and were scaled per 0.1 mL/min increment to facilitate clinical interpretation. Results were reported as odds ratios, 95% confidence intervals, and *p*-values.

A *p*-value < 0.05 was considered statistically significant.

## 3. Results

### 3.1. Characteristics of the Study Cohort

**Figure 2:**
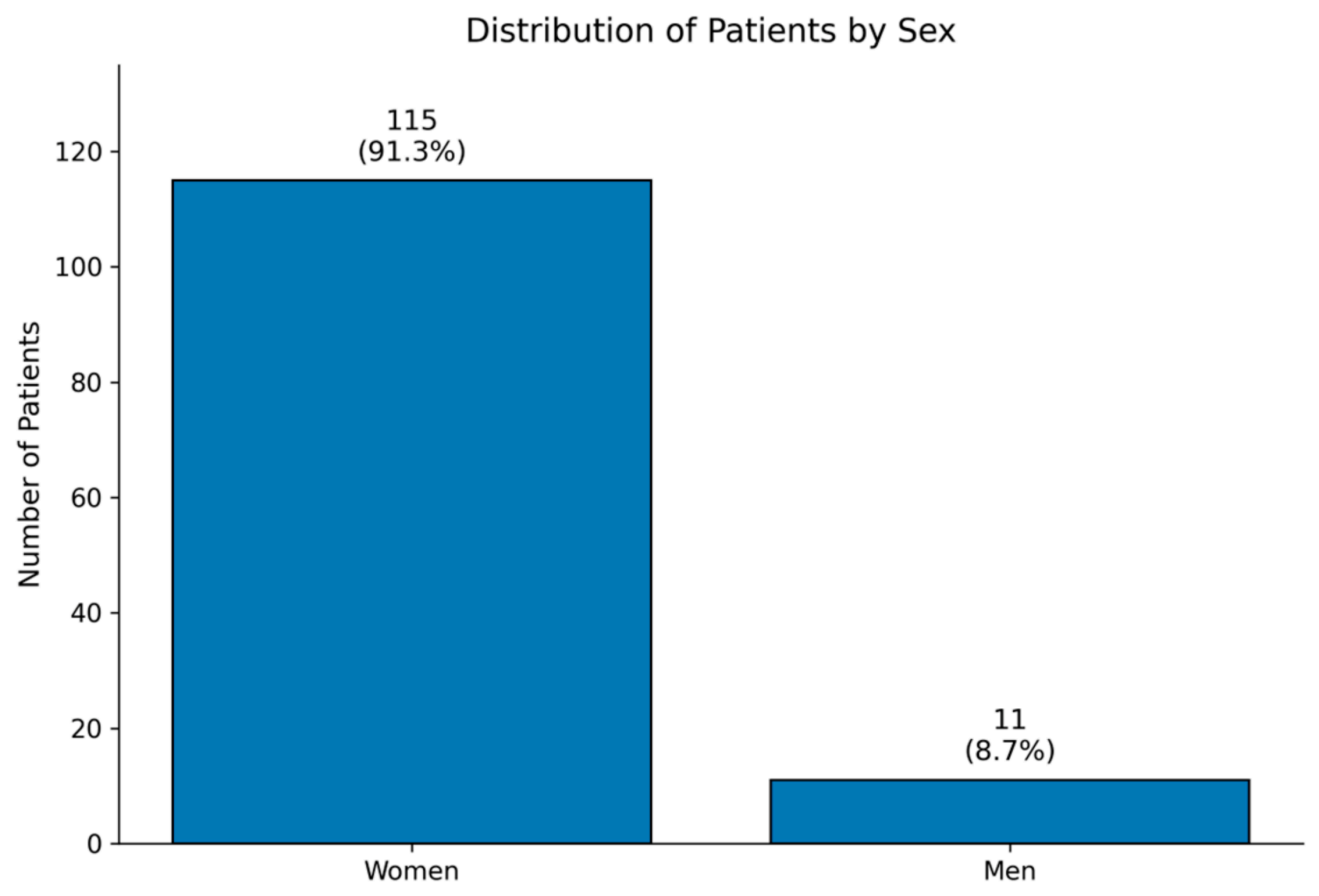
Distribution of Subjects by sex.

The study included 126 patients diagnosed with Sjögren’s syndrome. The mean age of the study population was 54.1 ± 13.3 years. Women represented the majority of the cohort, with 115 cases (91.3%), while men accounted for 11 cases (8.7%). The sex distribution of the study cohort is illustrated in Figure 2. Demographic characteristics of the study cohort are presented in Table 3.

### 3.2. Salivary Functional Assessment

**Figure 3:**
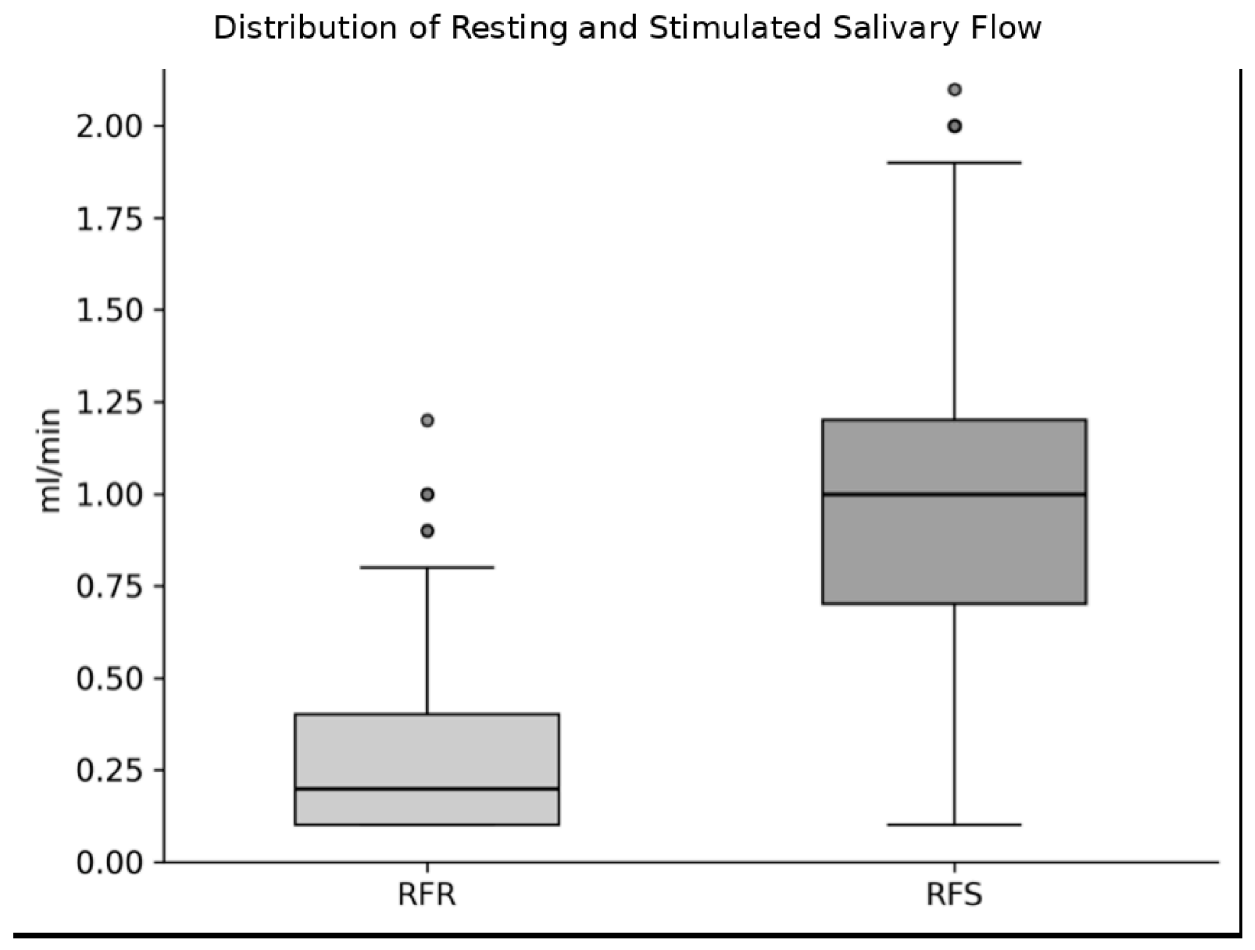
Distribution of resting and stimulated salivary flow values. The horizontal line within each box indicates the median, the lower and upper limits of the box represent the first and third quartiles, respectively, and the whiskers indicate values within 1.5 times the interquartile range. Circles represent outlier values.

The mean unstimulated whole salivary flow rate was 0.272 ± 0.246 mL/min, while the mean stimulated whole salivary flow rate was 1.04 ± 0.50 mL/min. The mean salivary pH was 6.38 ± 0.53. The main salivary parameters are summarized in Table 4. The distribution of unstimulated and stimulated salivary flow values is shown in Figure 3.

The mean unstimulated whole salivary flow rate was 0.270 ± 0.244 mL/min, with a median of 0.20 mL/min (IQR: 0.10–0.40). The mean stimulated whole salivary flow rate was 1.05 ± 0.50 mL/min, with a median of 1.00 mL/min (IQR: 0.70–1.30). The mean salivary pH was 6.39 ± 0.50, with a median of 6.40 (IQR: 6.20–7.00). The broad ranges of both salivary flow parameters indicated substantial functional heterogeneity within the cohort. The overall salivary dysfunction score is presented in Table 5.

The distribution of salivary dysfunction grades is presented in the table below (Table 6).

### 3.3. Unadjusted Comparison Between Primary and Secondary Sjögren’s Syndrome

In unadjusted between-group analyses, patients with primary Sjögren’s syndrome showed lower unstimulated and stimulated whole salivary flow rates than patients with secondary Sjögren’s syndrome.

Of the 126 patients included in the study, 44 were diagnosed with primary Sjögren’s syndrome and 82 with secondary Sjögren’s syndrome.

Patients with primary Sjögren’s syndrome had significantly lower unstimulated whole salivary flow compared with patients with secondary Sjögren’s syndrome (0.10 [0.10–0.20] vs. 0.20 [0.10–0.40] mL/min, *p* = 0.006). Similarly, stimulated whole salivary flow was significantly lower in the primary Sjögren’s syndrome group than in the secondary Sjögren’s syndrome group (0.70 [0.50–1.00] vs. 1.10 [0.90–1.40] mL/min, *p* = 0.0005).

No significant difference was observed between the two groups regarding salivary pH (*p* = 0.599). However, the distribution of salivary dysfunction severity differed significantly between primary and secondary Sjögren’s syndrome (*p* = 0.0013). These comparisons are summarized in Table 7.

**Figure 4:**
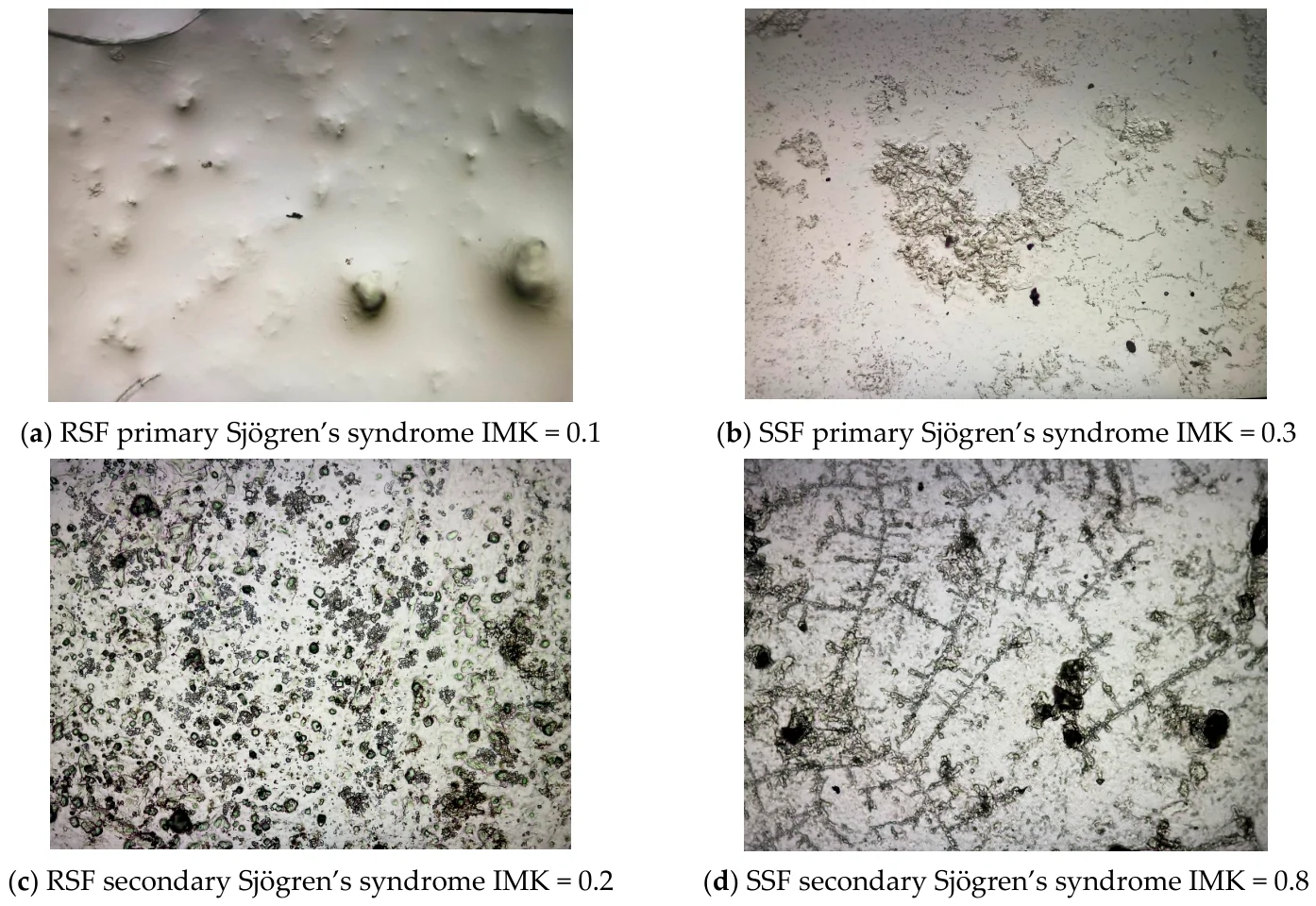
Representative salivary microcrystallization patterns in primary and secondary Sjögren’s syndrome: (**a**) unstimulated saliva from a patient with primary Sjögren’s syndrome, IMK-RFR = 0.1; (**b**) stimulated saliva from a patient with primary Sjögren’s syndrome, IMK-RFS = 0.3; (**c**) unstimulated saliva from a patient with secondary Sjögren’s syndrome, IMK-RFR = 0.2; and (**d**) stimulated saliva from a patient with secondary Sjögren’s syndrome, IMK-RFS = 0.8. Images were acquired using an Optika B-190TB digital optical microscope, 1000× (Optika S.r.l., Ponteranica, Italy), at 20× magnification. Lower IMK values indicate poorly represented or altered crystallization patterns, whereas higher IMK values indicate more preserved crystalline organization.

Representative salivary microcrystallization patterns from unstimulated and stimulated saliva samples in patients with primary and secondary Sjögren’s syndrome are shown in Figure 4.

### 3.4. Association Between Salivary Flow and Salivary Dysfunction Severity

Significant inverse correlations were identified between salivary flow parameters and salivary dysfunction grade. Unstimulated whole salivary flow showed a strong negative correlation with dysfunction grade, while stimulated whole salivary flow also showed a significant inverse correlation. These findings confirm the internal consistency of the functional grading system, as salivary dysfunction severity was defined using salivary functional parameters. Correlation results are presented in Table 8.

### 3.5. Exploratory Internal Consistency Analysis of Salivary Parameters

As an exploratory internal consistency assessment, ROC analysis was used to describe the alignment between individual salivary parameters and the predefined classification of grade ≥ 3 salivary dysfunction. Since dysfunction severity was partly derived from salivary flow and pH, the resulting AUC values should be interpreted as measures of internal consistency rather than as independent diagnostic or predictive performance.

Unstimulated whole salivary flow showed good internal discrimination of grade ≥ 3 salivary dysfunction, with an AUC of 0.826. Stimulated whole salivary flow showed very high internal discrimination, with an AUC of 0.998. Salivary pH showed limited internal discriminatory ability, with an AUC of 0.625.

Because standard maximum-likelihood logistic regression showed quasi-complete separation, Firth penalized logistic regression was used. In the multivariable model including both unstimulated and stimulated whole salivary flow, stimulated whole salivary flow remained associated with grade ≥ 3 salivary dysfunction. For stimulated whole salivary flow, the odds ratio per 0.1 mL/min increase was 0.033, 95% CI 0.002–0.557, *p* = 0.018. Unstimulated whole salivary flow was not associated with grade ≥ 3 dysfunction after adjustment for stimulated whole salivary flow, with an odds ratio per 0.1 mL/min increase of 0.392, 95% CI 0.051–3.032, *p* = 0.369.

Overall, these findings should be interpreted cautiously. Because salivary dysfunction severity was defined using functional salivary parameters, the ROC and regression results primarily reflect the internal coherence of the grading system and should not be regarded as independent diagnostic validation.

### 3.6. Salivary Microcrystallization Analysis

The mean IMK-RFR value in patients with Sjögren’s syndrome was 0.257 ± 0.108, while the mean IMK-RFS value was 0.471 ± 0.123. These values describe salivary microcrystallization patterns within the study cohort and were further analyzed according to sex and Sjögren’s syndrome subtype. Descriptive values of the microcrystallization indices are presented in Table 9.

No relevant differences in IMK values were observed according to sex. Patients with primary Sjögren’s syndrome had higher IMK-RFR values compared with patients with secondary Sjögren’s syndrome (0.293 ± 0.081 vs. 0.239 ± 0.116), while IMK-RFS values were similar between the two groups.

Inter-observer agreement for salivary microcrystallization assessment was evaluated in a randomly selected subset of 30 saliva samples independently assessed by two blinded evaluators. The agreement between evaluators was high for IMK-RFR, with an intraclass correlation coefficient of 0.86 (95% CI: 0.73–0.93), and for IMK-RFS, with an intraclass correlation coefficient of 0.89 (95% CI: 0.78–0.95). The overall intraclass correlation coefficient for IMK assessment was 0.88 (95% CI: 0.80–0.93), indicating good reproducibility of the microcrystallization evaluation.

Correlation analysis showed predominantly weak associations between salivary microcrystallization indices and the investigated clinical and functional variables. The only statistically significant association was observed between IMK-RFR and Sjögren’s syndrome subtype (Spearman’s rho = −0.256, *p* = 0.004), indicating a weak inverse relationship.

No significant correlations were identified between IMK indices and age, salivary flow parameters, salivary pH, sex, or salivary dysfunction severity. The detailed correlation analysis is presented in Table 9, while the strongest absolute Spearman correlations are summarized in Table 10.

The strongest absolute Spearman correlations between salivary microcrystallization indices and analyzed variables are presented in Table 11.

The only nominally significant association was observed between IMK-RFR and Sjögren’s syndrome subtype (Spearman’s rho = −0.256, nominal *p* = 0.004). However, after Holm–Bonferroni correction for the 16 exploratory Spearman correlations performed for IMK-RFR and IMK-RFS, this association did not remain statistically significant (adjusted *p* = 0.064).

## 4. Discussion

The present study evaluated salivary dysfunction in patients with primary and secondary Sjögren’s syndrome by integrating quantitative salivary flow measurements, salivary pH, and salivary microcrystallization indices. The main findings were that patients with primary Sjögren’s syndrome had significantly lower unstimulated and stimulated whole salivary flow rates than patients with secondary Sjögren’s syndrome, and that both salivary flow parameters were strongly and inversely associated with the severity of salivary dysfunction. In contrast, salivary pH showed limited discriminatory value, while salivary microcrystallization indices did not correlate robustly with salivary flow, pH, or dysfunction severity. These findings support the central role of salivary flow assessment in the objective evaluation of glandular dysfunction and suggest that salivary microcrystallization should currently be interpreted as an exploratory marker rather than as a substitute for functional salivary testing.

The demographic profile of the cohort was consistent with the established epidemiology of Sjögren’s syndrome, showing a marked female predominance and a mean age within the middle-adult range. Hormonal and sex-related immune mechanisms may contribute to this distribution. Estrogens and androgens modulate B-cell activity, autoantibody production, epithelial homeostasis, and inflammatory responses, while the hormonal changes occurring during the perimenopausal and postmenopausal periods may influence susceptibility to glandular dysfunction [4,17]. However, the relationship between sex hormones and Sjögren’s syndrome is complex and cannot fully explain the female predominance, which probably results from interactions among hormonal, genetic, epigenetic, and immune factors. Hormonal status was not evaluated in the present study; therefore, no direct conclusions regarding its contribution can be drawn from this cohort [18].

A relevant finding of this study was the more pronounced salivary impairment observed in patients with primary Sjögren’s syndrome. Both unstimulated and stimulated whole salivary flow rates were significantly lower in primary Sjögren’s syndrome compared with secondary Sjögren’s syndrome. This suggests that, in the present cohort, primary disease was associated with a more evident functional glandular phenotype. The finding is clinically plausible, as exocrine gland involvement is a defining feature of primary Sjögren’s syndrome [19,20]. However, this result should be interpreted cautiously, because the secondary Sjögren’s syndrome group may include patients with heterogeneous autoimmune backgrounds, disease durations, treatment exposures, and comorbidities, all of which may influence salivary gland function. Therefore, while the comparison between primary and secondary disease is clinically relevant, further studies with stratification according to the associated autoimmune disease are needed [21].

Although patients with primary Sjögren’s syndrome showed lower unstimulated and stimulated salivary flow rates than those with secondary Sjögren’s syndrome, these results should be interpreted cautiously. The comparisons were unadjusted, and factors such as age, sex, disease duration, associated autoimmune disease, comorbidities, and medication use may have influenced salivary gland function [22]. Therefore, these findings should be regarded as descriptive cohort-level differences rather than definitive evidence of independent disease-subtype effects.

The inverse correlations between salivary flow parameters and salivary dysfunction severity should be interpreted in the context of the grading system used in this study. Because dysfunction severity was defined using functional salivary parameters, these correlations primarily confirm the internal consistency of the classification rather than representing an independent validation of salivary flow as a severity marker. Nevertheless, the results support the clinical relevance of assessing both unstimulated and stimulated whole salivary flow, as these parameters describe complementary aspects of basal secretion and residual salivary gland functional reserve [23,24].

The ROC analysis indicated good discriminatory performance for unstimulated salivary flow and excellent discriminatory performance for stimulated salivary flow in identifying severe salivary dysfunction. The cut-off value of 0.1 mL/min for unstimulated salivary flow is consistent with the threshold included in the 2016 ACR/EULAR classification criteria for primary Sjögren’s syndrome [25]. Similarly, the 0.7 mL/min cut-off for stimulated salivary flow is clinically meaningful and compatible with values commonly used to indicate salivary hypofunction [13]. Nevertheless, these ROC findings must be interpreted with caution. In the present study, salivary dysfunction severity was defined using functional salivary parameters, which means that the high discriminatory performance of salivary flow partly reflects an internal mathematical relationship rather than an entirely independent validation. Therefore, these cut-off values should not be considered externally validated diagnostic thresholds based on the present data alone. Future studies should test their predictive value against independent clinical outcomes, such as oral complications, imaging findings, histopathological features, or patient-reported symptom burden [14,15,16,19].

In contrast to salivary flow parameters, salivary pH showed only limited discriminatory ability. Although salivary pH may provide useful information regarding the oral environment and potential risk for dental and mucosal complications, it did not behave as a strong marker of severe salivary dysfunction in this cohort. This finding is consistent with the fact that current diagnostic and classification approaches for Sjögren’s syndrome rely primarily on objective glandular tests, serology, histopathology, and increasingly imaging-based assessment, rather than salivary pH alone [13,26]. Its value may therefore be greater when integrated with oral clinical examination, caries risk assessment, microbiological factors, and dietary or medication-related variables.

The analysis of salivary microcrystallization represents the most exploratory component of this study. In the present cohort, IMK-RFR and IMK-RFS described the microcrystallization profile of patients with primary and secondary Sjögren’s syndrome. The lack of robust correlations between IMK indices and salivary flow, salivary pH, or dysfunction severity suggests that salivary microcrystallization does not simply mirror quantitative salivary secretion but may reflect qualitative physicochemical characteristics of saliva [4,17,22,23]. As no healthy control group was included, these findings should be interpreted as within-cohort observations and cannot establish differences between patients with Sjögren’s syndrome and individuals without the disease [27].

However, the internal correlation analysis did not show robust associations between IMK indices and salivary flow, salivary pH, or salivary dysfunction severity. The only statistically significant association was a weak inverse correlation between IMK-RFR and disease subtype. This indicates that salivary microcrystallization does not simply mirror quantitative salivary secretion. Rather, it may reflect a different biological dimension of saliva, related more to physicochemical composition than to volume. From this perspective, the absence of strong correlations with salivary flow should not necessarily be interpreted as a complete lack of biological relevance, but it does limit the immediate clinical applicability of IMK as an independent marker.

These findings have important implications. First, salivary microcrystallization cannot currently replace unstimulated or stimulated salivary flow measurement in the evaluation of Sjögren’s syndrome. Second, its potential role, if confirmed, is more likely to be complementary than diagnostic. Third, methodological standardization is essential before IMK can be considered for broader clinical or research use. Factors such as saliva collection conditions, slide preparation, drying time, ambient temperature and humidity, microscopy settings, scoring reproducibility, and inter-observer agreement may all influence the final microcrystallization score. Future studies should include larger and better-stratified primary and secondary Sjögren’s syndrome cohorts, standardized image acquisition, digital image analysis, and formal assessment of intra- and inter-observer reliability [28].

Moreover, the study was not designed to determine the reason why patients with Sjögren’s syndrome develop different degrees of oral disease or to evaluate the predictive value of salivary microcrystallization for oral health outcomes. Rather, its objective was to explore the relationship between salivary microcrystallization and established functional salivary parameters. Although no robust associations were identified, these findings help define the current limitations of IMK as a clinical biomarker and provide a basis for future prospective studies incorporating comprehensive oral health assessment and longitudinal clinical outcomes.

The present study has several limitations. First, it was a single-center, cross-sectional study, which limits generalizability and does not allow assessment of longitudinal changes in salivary function. Second, the secondary Sjögren’s syndrome group was not stratified according to the associated autoimmune disease, treatment exposure, disease duration, or comorbidities that may affect salivary secretion. Third, no healthy control group was included; therefore, the microcrystallization findings should be interpreted descriptively within the Sjögren’s syndrome cohort and comparatively between primary and secondary disease, rather than as evidence of differences versus healthy individuals. In addition, detailed oral health parameters, including periodontal status, dental caries experience, oral hygiene indices, oral mucosal lesions, and opportunistic oral infections, were not systematically recorded. Consequently, the present study cannot determine whether these factors influenced salivary microcrystallization patterns or functional salivary measurements. Future studies integrating comprehensive oral examinations with salivary functional assessment are warranted to better define the relationship between salivary dysfunction and oral health outcomes in Sjögren’s syndrome. The study protocol focused on salivary microcrystallization rather than cytological analysis, and the preparation method did not allow standardized assessment of epithelial cell quantity or morphology. Future studies combining microcrystallization with exfoliative cytology may better characterize mucosal alterations associated with salivary dysfunction in Sjögren’s syndrome. Moreover, oral mucosal colonization resistance was also not assessed because the protocol did not include standardized buccal epithelial sampling or microbiological evaluation. This may limit the mechanistic interpretation of the relationship between salivary dysfunction, mucosal defense, and oral microbial ecology [29]. Another limitation is represented by oral hygiene, which was not systematically evaluated and which may influence the observed salivary findings. Future studies should include standardized oral hygiene indices (such as OHI-S, Plaque Index, Silness-Löe, etc.) to better account for this potential confounding factor. Finally, the severity of salivary dysfunction was derived from functional salivary parameters, which introduces a degree of circularity in the correlation and ROC analyses involving salivary flow. The available samples were prepared specifically for salivary microcrystallization analysis and did not permit standardized retrospective assessment of epithelial cell desquamation or oral mucosal colonization resistance. These parameters should be prospectively investigated using dedicated cytological and microbiological protocols together with comprehensive oral health assessment.

Despite these limitations, the study provides clinically relevant information. It confirms the usefulness of simple salivary flow measurements in characterizing glandular dysfunction in Sjögren’s syndrome and highlights differences between primary and secondary disease. It also contributes preliminary data regarding salivary microcrystallization, suggesting that IMK may capture qualitative salivary alterations, but does not currently show sufficient correlation with functional impairment to support its use as an independent clinical marker. Further prospective, multicenter studies using independent clinical endpoints and standardized microcrystallization protocols are needed to clarify the incremental value of this method.

## 5. Conclusions

Salivary dysfunction was frequent in patients with Sjögren’s syndrome and was objectively reflected by reduced unstimulated and stimulated whole salivary flow rates. Both parameters were associated with salivary dysfunction severity, supporting the clinical value of salivary flow assessment as a simple, non-invasive method for evaluating glandular impairment.

In this cohort, patients with primary Sjögren’s syndrome showed lower unstimulated and stimulated salivary flow rates than patients with secondary Sjögren’s syndrome. However, because these comparisons were unadjusted, this finding should be interpreted as a descriptive cohort-level observation rather than as definitive evidence of an independent disease-subtype effect. Salivary pH showed limited discriminatory value and should be considered a complementary oral parameter rather than an independent marker of severe glandular dysfunction.

Salivary microcrystallization provided descriptive exploratory information regarding dried saliva patterns. However, IMK values were not robustly associated with salivary flow, salivary pH, or dysfunction severity. Therefore, IMK should not be interpreted as a diagnostic or severity marker at this stage.

Further prospective studies with larger and better-stratified primary and secondary Sjögren’s syndrome cohorts, standardized microcrystallization protocols, comprehensive oral health assessment, and independent clinical endpoints are needed to determine whether salivary microcrystallization provides incremental value in predicting clinically relevant oral outcomes.

## Figures and Tables

**Table 1:** Criteria used for grading salivary dysfunction severity.

Grade	Interpretation	Unstimulated Whole Salivary Flow	Stimulated Whole Salivary Flow	Salivary pH
1	Mild dysfunction	>0.30 mL/min	>1.00 mL/min	≥6.8
2	Moderate dysfunction	0.20–0.30 mL/min	0.80–1.00 mL/min	6.4–6.7
3	Moderate-to-severe dysfunction	0.10–0.19 mL/min	0.50–0.79 mL/min	6.0–6.3
4	Severe dysfunction	<0.10 mL/min	<0.50 mL/min	<6.0

The final salivary dysfunction grade was assigned according to the most severely impaired parameter. Thus, if at least one parameter fulfilled criteria for a higher dysfunction grade, the patient was classified in that higher grade.

**Table 2:** Classification of salivary microcrystallization index values according to the Leus method modified after Belskaya/Belischaia.

IMK Value	Interpretation
0.6–1.0	High microcrystallization
0.4–0.6	Moderate microcrystallization
0–0.4	Low microcrystallization

IMK, salivary microcrystallization index. Higher IMK values indicate a more preserved crystalline organization and greater salivary remineralization potential, whereas lower values indicate altered or poorly represented microcrystallization patterns.

**Table 3:** Demographic characteristics of the study cohort.

Variable	Value
Total number of patients	126
Age, years	54.1 ± 13.3
Female	115 (91.3%)
Male	11 (8.7%)

**Table 4:** Salivary functional parameters in the study cohort.

Parameter	*n*	Mean ± SD	Median [IQR]	Min–Max
Unstimulated whole salivary flow, mL/min	126	0.270 ± 0.244	0.20 [0.10–0.40]	0.10–1.20
Stimulated whole salivary flow, mL/min	126	1.05 ± 0.50	1.00 [0.70–1.30]	0.10–2.10
Salivary pH	126	6.39 ± 0.50	6.40 [6.20–6.80]	5.00–7.40

**Table 5:** Overall salivary dysfunction score.

Variable	*n*	Mean ± SD	Min	Max
Salivary dysfunction grade	126	2.03 ± 0.82	1	4

**Table 6:** Distribution of salivary dysfunction grades.

Grade	Interpretation	Number of Patients	Percentage	95% CI
1	Mild	36	28.6%	21.4–37.0%
2	Moderate	54	42.9%	34.6–51.6%
3	Moderate-to-severe	32	25.4%	18.6–33.6%
4	Severe	4	3.2%	1.2–7.9%

**Table 7:** Comparison of salivary parameters between patients with primary and secondary Sjögren’s syndrome. Data are expressed as median and interquartile range. Differences between groups were assessed using the Mann–Whitney U test.

Variable	Primary Sjögren’s Syndrome, *n* = 44 Median [IQR]	Secondary Sjögren’s Syndrome, *n* = 82 Median [IQR]	Difference in Medians	Effect Size r, Approximate 95% CI	*p*-Value
Unstimulated whole salivary flow, mL/min	0.10 [0.10–0.20]	0.20 [0.10–0.40]	−0.10	−0.245 [−0.402 to −0.073]	0.006
Stimulated whole salivary flow, mL/min	0.70 [0.50–1.00]	1.10 [0.90–1.40]	−0.40	−0.310 [−0.460 to −0.143]	0.0005
Salivary pH	6.40 [6.00–7.00]	6.40 [6.20–7.00]	0.00	0.047 [−0.129 to 0.220]	0.599
Salivary dysfunction grade	2.00 [1.00–2.00]	2.50 [1.00–3.00]	−0.50	−0.287 [−0.439 to −0.117]	0.0013

**Table 8:** Correlations between salivary flow parameters and salivary dysfunction severity.

Variable	Spearman’s Rho	*p*-Value
Unstimulated whole salivary flow vs. salivary dysfunction grade	−0.821	<0.001
Stimulated whole salivary flow vs. salivary dysfunction grade	−0.753	<0.001

**Table 9:** Descriptive analysis of salivary microcrystallization indices according to sex and Sjögren’s syndrome subtype.

Subgroup	Category	Variable	*n*	Mean	SD	Median	Min–Max
Sex	Female	IMK-RFR	115	0.257	0.111	0.3	0.100–0.500
Sex	Female	IMK-RFS	115	0.469	0.122	0.4	0.100–0.900
Sex	Male	IMK-RFR	11	0.264	0.081	0.3	0.100–0.400
Sex	Male	IMK-RFS	11	0.500	0.141	0.5	0.300–0.700
Disease subtype	Primary Sjögren’s syndrome	IMK-RFR	44	0.293	0.081	0.3	0.100–0.400
Disease subtype	Primary Sjögren’s syndrome	IMK-RFS	44	0.468	0.086	0.5	0.300–0.700
Disease subtype	Secondary Sjögren’s syndrome	IMK-RFR	82	0.239	0.116	0.2	0.100–0.500
Disease subtype	Secondary Sjögren’s syndrome	IMK-RFS	82	0.473	0.140	0.4	0.100–0.900

**Table 10:** Correlations between salivary microcrystallization indices and analyzed variables.

IMK Index	Correlated Variable	*n*	Pearson r	*p* Pearson	Spearman’s Rho	*p* Spearman	Significance
IMK-RFR	Age	126	−0.022	0.805	−0.034	0.710	Non-significant
IMK-RFR	Unstimulated salivary flow	126	−0.052	0.566	−0.100	0.269	Non-significant
IMK-RFR	Salivary pH	126	0.018	0.839	0.044	0.630	Non-significant
IMK-RFR	Stimulated salivary flow	126	0.012	0.899	0.005	0.954	Non-significant
IMK-RFR	CT	126	−0.080	0.376	−0.092	0.307	Non-significant
IMK-RFR	Salivary dysfunction grade	126	0.111	0.220	0.114	0.207	Non-significant
IMK-RFR	Sex	126	0.018	0.839	0.024	0.790	Non-significant
IMK-RFR	Sjögren’s syndrome subtype	126	−0.236	0.008	−0.256	0.004	Significant
IMK-RFS	Age	126	−0.102	0.254	−0.047	0.604	Non-significant
IMK-RFS	Unstimulated salivary flow	126	0.109	0.222	0.008	0.929	Non-significant
IMK-RFS	Salivary pH	126	0.058	0.520	0.112	0.212	Non-significant
IMK-RFS	Stimulated salivary flow	126	−0.043	0.631	−0.104	0.245	Non-significant
IMK-RFS	CT	126	0.001	0.990	−0.079	0.377	Non-significant
IMK-RFS	Salivary dysfunction grade	126	0.065	0.473	0.113	0.207	Non-significant
IMK-RFS	Sex	126	0.072	0.423	0.047	0.599	Non-significant
IMK-RFS	Sjögren’s syndrome subtype	126	0.019	0.829	−0.030	0.735	Non-significant

**Table 11:** Strongest absolute Spearman correlations between salivary microcrystallization indices and analyzed variables.

IMK Index	Variable	*n*	Spearman’s Rho	*p*-Value
IMK-RFR	Sjögren’s syndrome subtype	126	−0.256	0.004
IMK-RFR	Salivary dysfunction grade	126	0.114	0.207
IMK-RFS	Salivary dysfunction grade	126	0.113	0.207
IMK-RFS	Salivary pH	126	0.112	0.212
IMK-RFS	Stimulated salivary flow	126	−0.104	0.245
IMK-RFR	Unstimulated salivary flow	126	−0.100	0.269

## Data Availability

The original contributions presented in this study are included in the article. Further inquiries can be directed to the corresponding author.

## Abbreviations

The following abbreviations are used in this manuscript:

ACR	American College of Rheumatology
AUC	Area under the receiver operating characteristic curve
CI	Confidence interval
EULAR	European Alliance of Associations for Rheumatology
ICC	Intraclass correlation coefficient
IMK	Salivary microcrystallization index
IMK-RFR	Salivary microcrystallization index for unstimulated saliva
IMK-RFS	Salivary microcrystallization index for stimulated saliva
IQR	Interquartile range
OHI-S	Simplified Oral Hygiene Index
ROC	Receiver operating characteristic
SD	Standard deviation
